# Hyperoxia increases ventilator-induced lung injury via mitogen-activated protein kinases: a prospective, controlled animal experiment

**DOI:** 10.1186/cc5704

**Published:** 2007-02-22

**Authors:** Li-Fu Li, Shuen-Kuei Liao, Yu-Shien Ko, Cheng-Huei Lee, Deborah A Quinn

**Affiliations:** 1 Division of Pulmonary and Critical Care Medicine, Chang Gung Memorial Hospital, and Chang Gung University, 5 Fu-Hsing Street, Kweishan, Taoyuan 333, Taiwan; 2 Department of Respiratory Therapy, Chang Gung Memorial Hospital, 5 Fu-Hsing Street, Kweishan, Taoyuan 333, Taiwan; 3 Graduate Institute of Clinical Medical Sciences, Chang Gung University, 259 Wen-Hwa 1st Road, Kweishan, Taoyuan 333, Taiwan; 4 The First Cardiovascular Division, Department of Internal Medicine, Chang Gung Memorial Hospital, and Chang Gung University, 5 Fu-Hsing Street, Kweishan, Taoyuan 333, Taiwan; 5 Pulmonary and Critical Care Units, Department of Medicine, Massachusetts General Hospital, and Harvard Medical School, 55 Fruit Street, Boston, MA, USA

## Abstract

**Introduction:**

Large-tidal volume (V_T_) mechanical ventilation and hyperoxia used in patients with acute respiratory distress syndrome can damage pulmonary epithelial cells through lung inflammation and apoptotic cell death. Hyperoxia has been shown to increase ventilator-induced lung injury, but the mechanisms regulating interaction between large V_T _and hyperoxia are unclear. We hypothesized that the addition of hyperoxia to large-V_T _ventilation would increase neutrophil infiltration by upregulation of the cytokine macrophage inflammatory protein-2 (MIP-2) and would increase apoptosis via the mitogen-activated protein kinase pathways.

**Methods:**

C57BL/6 mice were exposed to high-V_T _(30 ml/kg) mechanical ventilation with room air or hyperoxia for one to five hours.

**Results:**

The addition of hyperoxia to high-V_T _ventilation augmented lung injury, as demonstrated by increased apoptotic cell death, neutrophil migration into the lung, MIP-2 production, MIP-2 mRNA expression, increased DNA binding activity of activator protein-1, increased microvascular permeability, and c-Jun NH_2_-terminal kinase (JNK) and extracellular signal-regulated kinase (ERK) 1/2 activation. Hyperoxia-induced augmentation of high-V_T_-induced lung injury was attenuated in JNK-deficient mice and in mice with pharmacologic inhibition of ERK activity by PD98059. However, only JNK-deficient mice, and not mice with ERK activity inhibition by PD98059, were protected from high-V_T_-induced lung injury without hyperoxia.

**Conclusion:**

We conclude that hyperoxia increased high-V_T_-induced cytokine production, neutrophil influx, and apoptotic cell death through activation of the JNK and ERK1/2 pathways.

## Introduction

Acute respiratory distress syndrome (ARDS) is an inhomogeneous lung disease characterized by non-cardiogenic pulmonary edema, release of cytokines, and influx of neutrophils and requires the use of mechanical ventilation with high levels of oxygen to adequately oxygenate the brain and other vital organs [1-14]. However, mechanical ventilation and prolonged exposure to hyperoxia can damage pulmonary epithelial cells through either apoptotic or non-apoptotic cell death [1-10]. Mechanical ventilation with high tidal volume (V_T_) values causes acute lung injury (ventilator-induced lung injury [VILI]) characterized by an inflammatory response that is similar to that caused by bacterial lipopolysaccharide [11,12] and is dependent on activation of extracellular signal-regulated kinase (ERK) 1/2 and c-Jun NH_2_-terminal kinase (JNK), but not p38 [10]. Both large-V_T _ventilation and hyperoxia alone can lead to the production of inflammatory cytokines, including tumor necrosis factor-alpha, interleukin (IL)-1β, and murine macrophage inflammatory protein-2 (MIP-2), a functional homolog of human IL-8 in rodents [7,12,14,15].

The effects of hyperoxia on the lung have long been recognized. Mice exposed to hyperoxia develop a condition similar to ARDS, which is dependent on an increased production of reactive oxygen species by mitochondria [13,16]. Hyperoxia has been shown to cause alveolar hyaline membrane formation, edema, hyperplasia, proliferation of type II alveolar epithelial cells, destruction of type I alveolar epithelial cells, interstitial fibrosis, and pulmonary vascular remodeling [7]. Hyperoxia has been shown to include activation of all three major mitogen-activated protein kinase (MAPK) pathways – ERK1/2, JNK, and p38 – in various experimental models [6,8,17]. In previous studies, hyperoxia significantly exacerbated large-V_T _VILI but the interaction between them was unclear [3,7,9]. We hypothesized that the addition of hyperoxia to large-V_T _ventilation would increase MIP-2 production, neutrophil infiltration, and apoptosis via the MAPK pathways.

## Materials and methods

### Experimental animals

Male C57BL/6 mice (wild-type JNK^+/+^, JNK1^-/-^, or JNK2^-/- ^on a C57BL/6 background) weighing between 20 and 25 g were obtained from The Jackson Laboratory (Bar Harbor, ME, USA) and the National Laboratory Animal Center (Taipei, Taiwan) [18,19]. JNK knockout mice develop normally, have no known abnormal pathology, are fertile, are of normal size, and have normal lung parenchyma and airways [20]. This study was performed in accordance with animal experimental guidelines of the National Institutes of Health (NIH) (Bethesda, MD, USA) and with approval of the local research committee.

### Ventilator protocol

We used our established mouse model of VILI as previously described [20]. In brief, mice were ventilated with 30 ml/kg at 65 breaths per minute for one and five hours while breathing room air or hyperoxia (> 95% oxygen). Oxygen was fed into the inspiratory port of the ventilator when needed. Spontaneously breathing animals were exposed to hyperoxia in an enclosed chamber as previously described [7]. Our previous work has shown that activation of MAPK and increased mRNA expression occurred approximately one hour after cell stretch, whereas changes in cytokine production and neutrophil infiltration occur later. One hour of high-V_T _ventilation was used to collect samples of total RNA and protein for Western blot analysis of MAPKs, and five hours of ventilation was used for collection of samples of MIP-2, myeloperoxidase (MPO), Evans blue dye (EBD) leak [20], and apoptotic and immunohistochemical analyses. At the end of the study period, heparinized blood was taken from the arterial line for analysis of arterial blood gas and the mice were sacrificed. After sacrifice, the lungs were lavaged three times with 0.6 ml of 0.9% normal saline. The effluents were pooled and centrifuged at 2,000 rpm for 10 minutes. Supernatants were frozen at -80°C for further analysis of cytokine. For histopathology, the lungs were removed *en bloc*, and polyethylene tubing was inserted into the trachea. The lungs were filled with 10% neutral buffered formalin (pH 6.8 to 7.2) at a pressure of 30 cm H_2_O and stored in formalin. For measurement of kinase activation, the lungs were homogenized in 3 ml of lysis buffer (20 mM HEPES [pH 7.4], 1% Triton X-100, 10% glycerol, 2 mM ethylene glycol-bis [β-aminoethyl ether]-N,N,N',N'-tetraacetic acid, 50 μM β-glycerophosphate, 1 mM sodium orthovanadate, 1 mM dithiotreitol, 400 μM aprotinin, and 400 μM phenylmethylsulfonyl fluoride), transferred to Eppendorf tubes, and placed on ice for 15 minutes. Tubes were centrifuged at 14,000 rpm for 10 minutes at 4°C, and supernatant was flash-frozen. For isolation of total RNA, the lungs were homogenized in 1.5 ml of TRIzol reagent and isolated according to the manufacturer's directions (Invitrogen Corporation, Carlsbad, CA, USA) and flash-frozen. The experimental design of this study is summarized in Table 1.

**Table 1:** Experimental design and numbers of animals per group

	Control (WT, JNK KO)	Control + O2 (WT, JNK KO)	V_T _of 30 ml/kg	V_T _of 30 ml/kg + O2	V_T _of 30 ml/kg + JNK1 KO	V_T _of 30 ml/kg + JNK1 KO + O2	V_T _of 30 ml/kg + ERK inhibitor	V_T _of 30 ml/kg + ERK inhibitor + O2
MPO, MIP-2 (5 hours of ventilation)	5	5	5	5	5	5	5	5
JNK, p38, ERK (1 hour of ventilation)	5	5	5	5	5	5	5	5
PARP (1, 2, and 5 hours of ventilation)	5	5	5	5	5	5	5	5
MIP-2 mRNA (1 hour of ventilation)	5	5	5	5	5	5	5	5
EBD assay (5 hours of ventilation)	5	5	5	5	5	5	5	5
AP-1, NF-κB (1 hour of ventilation)	5	5	5	5	5	5	5	5
IHC assay (5 hours of ventilation)	5	5	5	5	5	5	5	5
TUNEL assay (5 hours of ventilation)	5	5	5	5				
Electron microscopy	2	2	2	2				

AP-1 and NF-κB binding; total expression and phosphorylated forms of JNK, p38, and ERK1/2; PARP; IHC; and MIP-2 mRNA expression were measured in lung tissue. Time points for measurements were determined according to our previous findings that mediator activation occurs early in ventilator-induced lung injury and neutrophil infiltration occurs later [20]. AP-1, activator protein-1; Control, spontaneously breathing, non-ventilated mice; EBD, Evans blue dye (a measurement of microvascular leak in the lung); ERK, extracellular signal-regulated kinase; IHC, immunohistochemical stain; JNK, c-Jun NH_2_-terminal kinase; KO, knockout; MIP-2, macrophage inflammatory protein-2 (a functional homolog of IL-8); MPO, myeloperoxidase (a measurement of total neutrophil infiltration in the lung); NF-κB, nuclear factor-kappa-B; O2, hyperoxia; PARP, poly (ADP-ribose) polymerase; TUNEL, terminal deoxynucleotidyl transferase-mediated dUTP-biotin nick end-labeling, a measurement of apoptotic cells in the lung; V_T_, tidal volume; WT, wild-type.

### EBD analysis

Extravasation of EBD (Sigma-Aldrich, St. Louis, MO, USA) into the interstitium was used as a quantitative measure of changes of microvascular permeability in acute lung injury [20]. Thirty minutes before the end of mechanical ventilation, 30 mg/kg of EBD was injected through the internal jugular vein. At the time of sacrifice (after five hours of mechanical ventilation), the lungs were perfused free of blood by means of 1 ml of 0.9% normal saline via the right ventricle and removed *en bloc*. EBD was extracted from lung tissue after homogenization for two minutes in 5 ml of formamide (Sigma-Aldrich) and incubated at 37°C overnight. The supernatant was separated by centrifugation at 5,000 *g *for 30 minutes, and the amount was recorded. EBD in the plasma and lung tissue was quantitated by dual-wavelength spectrophotometric analysis at 620 and 740 nm. The method corrects the specimen's absorbance at 620 nm for the absorbance of contaminating heme pigments by means of the following formula: corrected absorbance at 620 = actual absorbance at 620 nm - (1.426 [absorbance at 740 nm] + 0.03). We calculated the EBD amount extracted from lung tissue and divided the amount by the weight of the lung tissue.

### Measurement of MIP-2

MIP-2 (1 pg/ml) was measured in bronchoalveolar lavage (BAL) fluid by means of a commercially available immunoassay kit containing antibodies that were cross-reactive with rat and mouse MIP-2 (Biosource International, Camarillo, CA, USA). Each sample was run in duplicate according to the manufacturer's instructions.

### MPO assay

The lungs (0.12 to 0.17 g) were homogenized in 5 ml of phosphate buffer (20 mM, pH 7.4). One milliliter of the homogenate was centrifuged at 10,000 *g *for 10 minutes at 4°C. The resulting pellet was resuspended in 1 ml of phosphate buffer (50 mM, pH 6.0) containing 0.5% hexadecyltrimethylammonium bromide. The suspension was then subjected to three cycles of freezing (on dry ice) and thawing (at room temperature), after which it was sonicated for 40 seconds and centrifuged again at 10,000 *g *for 5 minutes at 4°C. The supernatant was assayed for MPO activity by measuring the hydrogen peroxide (H_2_O_2_)-dependent oxidation of 3,3', 5,5'-tetramethylbenzidine (TMB). In its oxidized form, TMB has a blue color, which was measured spectrophotometrically at 650 nm. The reaction mixture for analysis consisted of 25 μl of tissue sample, 25 μl of TMB (final concentration 0.16 mM) dissolved in dimethylsulfoxide, and 200 μl of H_2_O_2 _(final concentration 0.30 mM) dissolved in phosphate buffer (0.08 M, pH 5.4) minutes prior to being added to the mixture. The reaction mixture was incubated for 3 minutes at 37°C, and the reaction was stopped by adding 1 ml of sodium acetate (0.2 M, pH 3.0), after which absorbance at 650 nm was measured. The absorbance (A650) was reported in units of optical density per gram of wet lung weight.

### Extraction of nuclear proteins and electrophoretic mobility shift assay

The lungs (0.12 to 0.17 g) were homogenized and extraction of nuclear protein was performed as previously described [21]. Electrophoretic mobility shift assay (EMSA) was performed with a digoxigenin (DIG) Gel Shift Kit according to instructions of the manufacturer (Roche Diagnostics GmbH, Mannheim, Germany). Briefly, nucleoproteins (10 μg) were incubated with DIG-labeling probe in a 10-μl reaction mixture containing 1× labeling buffer, 5 mM CoCl_2_, 0.05 mM DIG-ddUTP, and 20 U/μl terminal transferase. The oligonucleotide sequences were as follows: activator protein-1 (AP-1) oligonucleotide (5'-d [C GCTTGATGAGTCAG-CCGGAA]-3') and nuclear factor-kappa-B (NF-κB) oligonucleotide (5'-AGTTGAGGGGACTTTCCCAGG-3') (Santa Cruz Biotechnology, Inc., Santa Cruz, CA, USA). To complete the specific binding reactions, each of the 100-fold molar excesses of unlabeled oligonucleotides (negative control) was added to the binding mixture before addition of the labeled probe. Nucleoprotein complexes were resolved on an 8% non-denaturing polyacrylamide gel and were electrotransferred to nylon membrane (Amersham Pharmacia Biotech, now part of GE Healthcare, Little Chalfont, Buckinghamshire, UK). Blots were blocked with 1% dried milk in maleic acid buffer and incubated with anti-DIG-alkaline phosphatase (1:10,000). Blots were developed by 3-(4-methoxyspiro{1,2-dioxetane-3,2'-(5'-chloro)tricyclo [3.3.1.1^3,7^] decan}-4-yl)phenyl phosphate and exposed to Kodak XAP-5 film (Eastman Kodak, Rochester, NY, USA) at room temperature.

### Immunoblot analysis

Crude cell lysates were matched for protein concentration, resolved on a 10% bis-acrylamide gel, and electrotransferred to Immobilon-P membranes (Millipore Corporation, Billerica, MA, USA). For assay of total JNK, p38, and ERK1/2 phosphorylation protein expression, Western blot analyses were performed with antibodies to phospho-JNK, phospho-p38, and phospho-ERK1/2 (New England Biolabs, Inc., Ipswich, MA, USA). For determination of total JNK, p38, ERK1/2 protein expression, Western blot analyses were performed with the respective antibodies (Santa Cruz Biotechnology, Inc.). Blots were developed by enhanced chemiluminescence (NEN Life Science Products, now part of PerkinElmer Life and Analytical Sciences, Inc., Waltham, USA).

### Immunohistochemistry

The lung tissues from control, non-ventilated mice exposed to high-V_T _ventilation for five hours while breathing room air or hyperoxia were paraffin-embedded, sliced at 4 μm, deparaffinized, antigen-unmasked in 10 mM sodium citrate (pH 6.0), and incubated with phospho-JNK, phospho-ERK1/2, or poly(ADP-ribose)polymerase (PARP) primary antibody (1:100; New England Biolabs, Inc.) and biotinylated goat anti-rabbit secondary antibody (1:100) according to the instructions of an immunohistochemical kit (Santa Cruz Biotechnology, Inc.). The specimens were further conjugated with horseradish peroxidase-streptoavidin complex, detected by diaminobenzidine (DAB) substrate mixture, and counterstained by hematoxylin. A dark brown DAB signal indicated positive staining of MAPKs of epithelial cells, and shades of light blue signified non-reactive cells.

### Electron microscopy assay

The lungs were filled with 3% glutaraldehyde in 0.1 M cacodylate buffer (pH 7.4) at a pressure of 30 cm H_2_O and fixed in the same glutaraldehyde buffer for one hour at 4°C. The lung tissues were then postfixed in 1% osmium tetroxide (pH 7.4), dehydrated in a graded series of ethanol, and embedded in EPON-812. Thin sections (70 nm) were cut, stained with uranyl acetate and lead citrate, and examined on a Hitachi H-7500 EM transmission electron microscope (Hitachi, Ltd., Tokyo, Japan).

### Pharmacologic inhibitor

ERK1/2 inhibitor (PD-98059; Calbiochem, San Diego, CA, USA), which has been shown to be useful in elucidating the role of ERK [22], was given subcutaneously (1 mg/kg) 30 minutes before ventilation. The dose was based on previous *in vivo *studies that showed that 1 mg/kg inhibited ERK1/2 activity [23].

### Statistical evaluation

The MIP-2 mRNA, EMSA, and Western blots were quantitated using an NIH image analyzer ImageJ 1.27z (NIH) and presented as the ratio of phospho-MAPK to MAPK (relative phosphorylation) in arbitrary units. Values were expressed as the mean ± standard error of the mean for at least five experiments. The data of neutrophils, MIP-2, MPO, EBD, terminal deoxynucleotidyl transferase-mediated dUTP-biotin nick end-labeling (TUNEL) analysis, and immunohistochemical analysis were analyzed using Statview 5.0 Abascus Concepts Inc., Cary, NC, USA, and SAS Institute Inc., Cary, NC, USA). All results of Western blot, EMSA, and MPO were normalized to control, non-ventilated mice breathing room air. Analysis of variance was used to assess the statistical significance of the differences, followed by multiple comparisons with a Scheffe's test, and a *p *value of less than 0.05 was considered statistically significant. Additional details, including the experimental design and the number of animals used in each group, RNase protection assay, and TUNEL assay, were the same as those previously described [17].

## Results

### Physiologic data

As we previously published [20], there were no statistical differences in pH, PaCO_2 _(arterial carbon dioxide tension), or mean arterial pressure from the beginning to the end of mechanical ventilation. Inhibition of JNK activation with JNK1 knockout mice reduced the effects of hyperoxia on high-V_T_-induced infiltration of neutrophils, cytokine production, and microvascular permeability.

**Figure 1:**
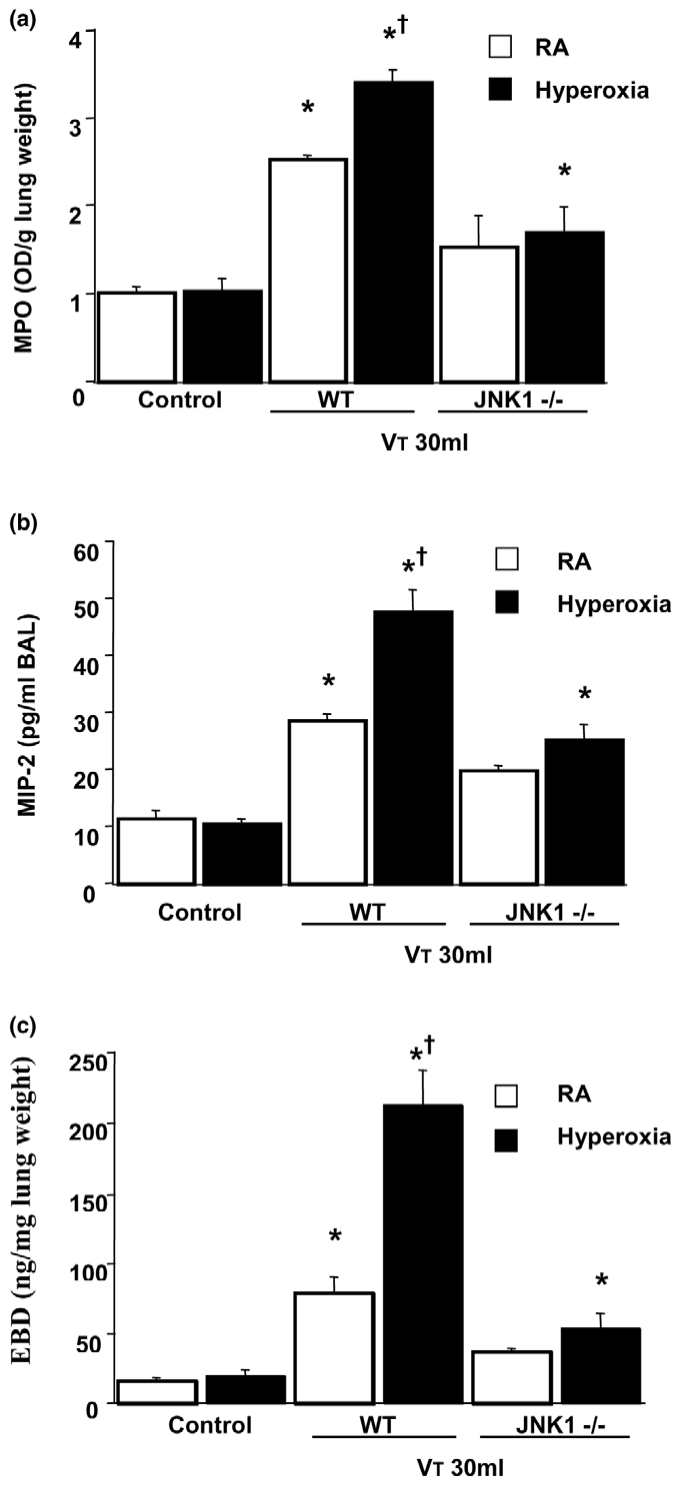
Effects of hyperoxia on stretch-induced neutrophil infiltration, macrophage inflammatory protein-2 (MIP-2) production, and microvascular leak in JNK knockout mice. **(a) **Myeloperoxidase (MPO) assay of lung tissue from control, non-ventilated mice, and mice ventilated for five hours at a tidal volume (V_T_) of 30 ml/kg with room air (RA) or hyperoxia (*n *= 5 per group). **(b) **MIP-2 production in bronchoalveolar lavage (BAL) fluid from control, non-ventilated mice, and mice ventilated for five hours at a V_T _of 30 ml/kg with RA or hyperoxia (*n *= 5 per group). **(c) **Evans blue dye (EBD) analysis of lung tissue from control, non-ventilated mice, and mice ventilated for five hours at a V_T _of 30 ml/kg with RA or hyperoxia (*n *= 5 per group). *p < 0.05 versus control, non-ventilated mice; †*p *< 0.05 versus all other groups. JNK, c-Jun NH_2_-terminal kinase; OD, optical density; WT, wild-type.

To determine the effects of hyperoxia on neutrophil content in the vasculature, lung parenchyma, and alveoli, we measured MPO activity (Figure 1a). The MPO levels in mice ventilated with hyperoxia at a V_T _of 30 ml/kg were significantly elevated compared with control, non-ventilated mice and mice ventilated with room air at a V_T _of 30 ml/kg. By using JNK1^-/- ^knockout mice receiving room air or hyperoxia with high-V_T _(30 ml/kg) mechanical ventilation, we found significantly decreased levels of MPO in JNK knockout mice.

**Figure 2:**
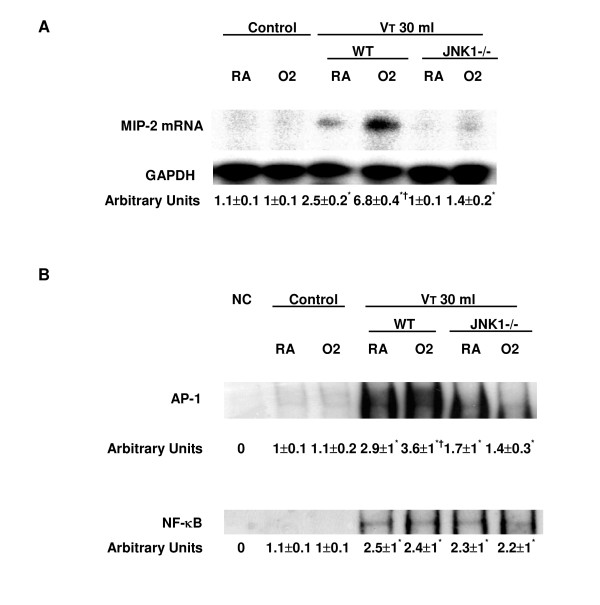
Effects of hyperoxia on stretch-induced macrophage inflammatory protein-2 (MIP-2) mRNA expression and DNA binding in JNK knockout mice. **(a) **MIP-2 mRNA (top panel), GAPDH mRNA (middle panel), and arbitrary units (bottom panel) from control, non-ventilated mice, and mice ventilated at a tidal volume (V_T_) 30 ml/kg for one hour with room air (RA) or hyperoxia (*n *= 5 per group). The ratio of MIP-2 mRNA to GAPDH was expressed in arbitrary units. The expression of MIP-2 mRNA was performed by RNase protection assay. **(b) **Nuclear proteins of lung tissue from control, non-ventilated mice, and mice ventilated at a V_T _of 30 ml/kg without or with hyperoxia were incubated with or without excess unlabeled activator protein-1 (AP-1) or nuclear factor-kappa-B (NF-κB) consensus oligonucleotide (negative control) (*n *= 5 per group). Arbitrary units were expressed as increase of DNA-binding activity as described in Materials and methods. *p < 0.05 versus control, non-ventilated mice; †*p *< 0.05 versus all other groups. GAPDH, glyceraldehyde-3-phosphate dehydrogenase; JNK, c-Jun NH_2_-terminal kinase; NC, control, non-ventilated mice; O2, mice with hyperoxia; WT, wild-type.

To determine whether the increased neutrophil sequestration with hyperoxia in high-V_T _mechanical ventilation was associated with upregulation of chemotactic factors for neutrophils and increased AP-1 DNA binding, we measured MIP-2 mRNA expression, AP-1-binding activity, and MIP-2 protein production for one and five hours of mechanical ventilation, respectively (Figures 1b and 2a). We used EBD to measure microvascular permeability (Figure 1c). We found that hyperoxia increased ventilator-induced MIP-2 mRNA expression, AP-1 DNA-binding activity, MIP-2 protein production, and EBD leak in the mice ventilated at a V_T _of 30 ml/kg compared with those of control, non-ventilated mice and mice ventilated with room air at a V_T _of 30 ml/kg. The effects of hyperoxia on high-V_T _(30 ml/kg) ventilator-induced expression of MIP-2 mRNA, AP-1-binding activity, production of MIP-2 protein, and EBD leak were significantly reduced via homozygous disruption of the JNK1 or JNK2 locus (data not shown). In a previous study, we found similar reductions in MPO and MIP-2 in JNK1 and JNK2 knockout mice, suggesting that sequential activation of JNK1 and JNK2 may be needed for activation of the pathway [20]. The NF-κB-binding activity was also increased, but there were no significant differences between the mice ventilated with room air and hyperoxia (Figure 2b). This suggested that addition of oxygen may be involved in high-V_T_-induced MIP-2 production and that MIP-2 production was dependent, in part, on the JNK/AP-1 pathway.

**Figure 3:**
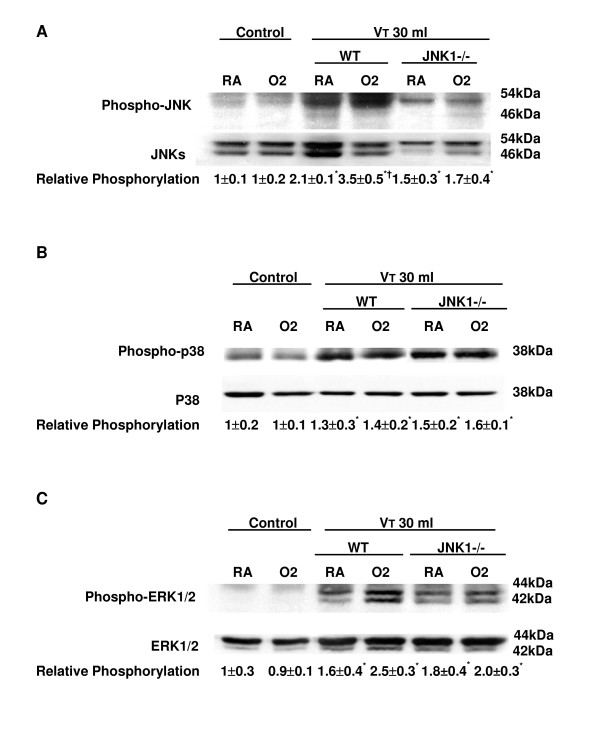
Effects of hyperoxia on high-tidal volume (V_T_)-induced mitogen-activated protein kinase activation. **(a) **Phosphorylated JNK expression (top panel), total JNK protein expression (middle panel), and quantitation by arbitrary units (bottom panel) (*n *= 5 per group). **(b) **Phosphorylated p38 expression (top panel), total p38 protein expression (middle panel), and quantitation by arbitrary units (bottom panel) (*n *= 5 per group). **(c) **Phosphorylated ERK1/2 expression (top panel), total ERK1/2 protein expression (middle panel), and quantitation by arbitrary units (bottom panel) (*n *= 5 per group). The relative phosphorylation was expressed in arbitrary units. *p < 0.05 versus control, non-ventilated mice; †*p *< 0.05 versus all other groups. ERK, extracellular signal-regulated kinase; JNK, c-Jun NH_2_-terminal kinase; O2, mice with hyperoxia; RA, mice with room air; WT, wild-type C57BL/6 mice.

To further define the effects of hyperoxia on MAPK activation in VILI, we measured the activity of three members of the MAPK families – JNKs, p38, and ERK1/2 – in mice exposed to high-V_T _(30 ml/kg) mechanical ventilation for one hour while breathing room air or hyperoxia. Hyperoxia increased the high-V_T_-induced phosphorylation of JNKs and ERK1/2 but did not change the expression of total non-phosphorylated proteins of JNKs and ERK1/2 (Figure 3a,c). Both JNK1 and JNK2 phosphorylation decreased in JNK1 knockout mice. JNK1 knockout mice have an intact JNK2 gene. However, each gene was capable of making the various isoforms of JNK via alternate splicing [20], and this may have led to a compensatory decrease in JNK2 expression.

**Figure 4:**
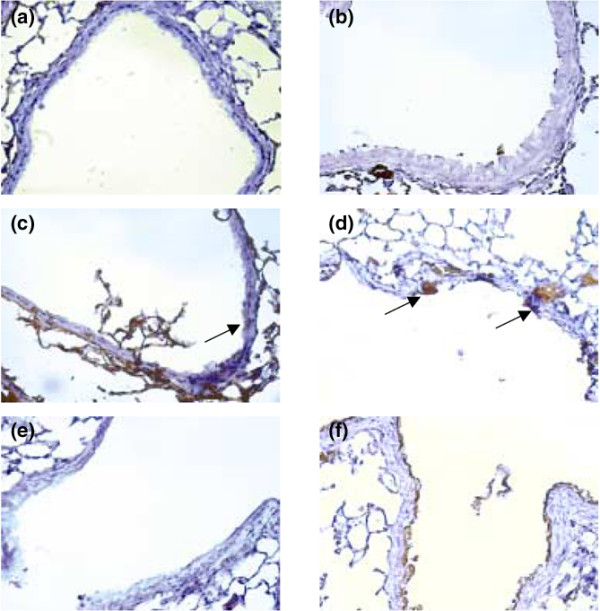
Effects of hyperoxia on stretch-induced JNK activation of airway epithelium in JNK knockout mice. Representative photomicrographs (×400) with phospho-JNK staining of the lung sections after five hours of mechanical ventilation with room air or hyperoxia (*n *= 5 per group). **(a) **Control wild-type mice with room air. **(b) **Control wild-type mice with hyperoxia. **(c) **Wild-type mice ventilated at a tidal volume (V_T_) of 30 ml/kg with room air. **(d) **Wild-type mice ventilated at a V_T _of 30 ml/kg with hyperoxia. **(e) **JNK1^-/- ^mice ventilated at a V_T _of 30 ml/kg with room air. **(f) **JNK1^-/- ^mice ventilated at a V_T _of 30 ml/kg with hyperoxia. Positive staining of airway epithelia is identified by arrows. A dark brown DAB signal indicates positive staining for JNK in the lung epithelium, and shades of bluish tan signify non-reactive cells. DAB, diaminobenzidine; JNK, c-Jun NH_2_-terminal kinase.

**Figure 5:**
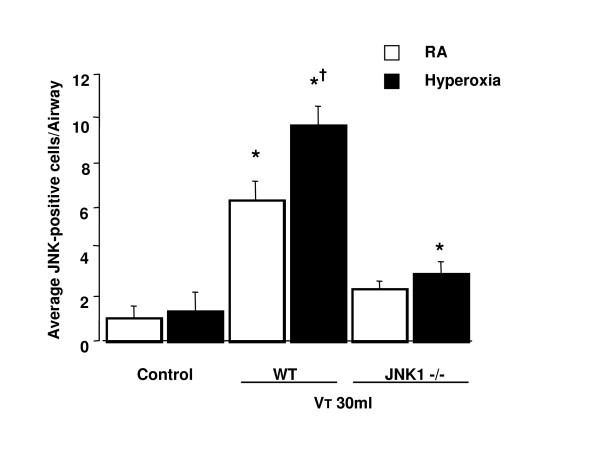
Effects of hyperoxia on stretch-induced JNK activation of airway epithelium in JNK knockout mice. Phospho-JNK-positive cells were quantified as the average number of epithelial cells with dark brown DAB signals per bronchiole, which were counted from 10 randomly chosen bronchioles of each section (*n *= 5 per group). *p < 0.05 versus control, non-ventilated mice; †*p *< 0.05 versus all other groups. DAB, diaminobenzidine; JNK, c-Jun NH_2_-terminal kinase; RA, room air; V_T_, tidal volume; WT, wild-type mice.

The effects of hyperoxia were significantly reduced in homozygous JNK knockout mice ventilated at a V_T _of 30 ml/kg. The phosphorylation of p38 was also increased, but there was no significant difference between high-V_T_-ventilated mice with or without hyperoxia in wild-type or JNK knockout mice (Figure 3b). Using immunohistochemistry, we confirmed the results of Western blot (Figure 3). Hyperoxia increased positive staining of phospho-JNK in airway epithelium of mice ventilated at a V_T _of 30 ml/kg for five hours (Figures 4 and 5). The increases of positive staining of phospho-JNK on airway epithelium were reduced in homozygous JNK knockout mice. Due to poor breeding performance of homozygous JNK2^-/- ^mice, only a few experiments were carried out with this strain and these experiments showed a trend similar to that of JNK1^-/- ^mice (data not shown). These data added further evidence that hyperoxia-augmented lung stretch-induced lung inflammation was dependent, in part, on the JNK pathway, but the role of ERK activation needed to be further explored. Inhibition of ERK1/2 activation with PD98059 reduced the effects of hyperoxia on high-V_T_-induced infiltration of neutrophils, MIP-2 protein production, and microvascular permeability.

**Figure 6:**
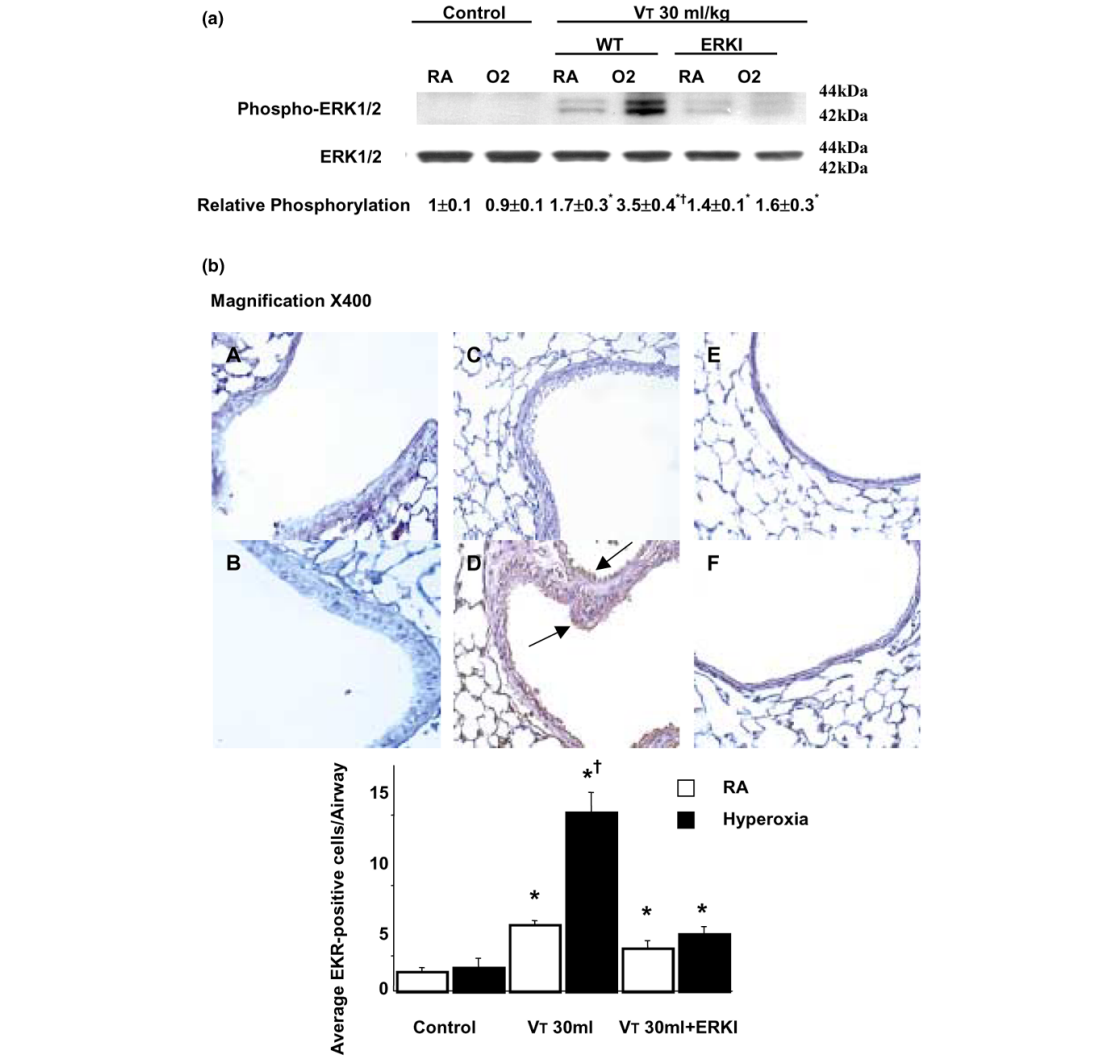
PD98059 reduced the effects of hyperoxia on high-tidal volume (V_T_)-induced ERK1/2 activation. The mice ventilated at a V_T _of 30 ml/kg (VT 30 ml) with or without hyperoxia were pretreated with 1 mg/kg PD98059 for 30 minutes. **(a) **Phosphorylated ERK1/2 expression (top panel), total ERK1/2 protein expression (middle panel), and quantitation by arbitrary units (bottom panel) (*n *= 5 per group). The relative phosphorylation was expressed in arbitrary units. **(b) **Representative photomicrographs (×400) with phospho-ERK1/2 staining of the lung sections (*n *= 5 per group). **(A) **Control wild-type mice with room air. **(B) **Control wild-type mice with hyperoxia. **(C) **Wild-type mice ventilated at a V_T _of 30 ml/kg with room air. **(D) **Wild-type mice ventilated at a V_T _of 30 ml/kg with hyperoxia. **(E) **Mice pretreated with PD98059 and ventilated at a V_T _of 30 ml/kg with room air. Positive staining of airway epithelia is identified by arrows. **(F) **Mice pretreated with PD98059 and ventilated at a V_T _of 30 ml/kg with hyperoxia. Phospho-ERK1/2-positive cells were quantified as the average number of epithelial cells with dark brown DAB signals per bronchiole, which were counted from 10 randomly chosen bronchioles of each section (*n *= 5 per group). *p < 0.05 versus control, non-ventilated mice; †*p *< 0.05 versus all other groups. DAB, diaminobenzidine; ERK, extracellular signal-regulated kinase; O2, mice with hyperoxia; RA, mice with room air; WT, wild-type mice.

To determine the role of ERK1/2 activation on the effects of hyperoxia in VILI, we measured the activity of ERK1/2 in mice pretreated with PD98059 and exposed to high-V_T _(30 ml/kg) mechanical ventilation for one hour while breathing room air or hyperoxia (Figure 6). There was a significant inhibition of ERK1/2 phosphorylation but no change in the expression of total non-phosphorylated ERK1/2 protein in mice with room air and hyperoxia (> 95% oxygen). ERK1 and ERK2 were affected equally. Using immunohistochemistry (Figure 6b), we confirmed the results of Western blot (Figure 6a). Hyperoxia increased the positive staining of phospho-ERK1/2 in airway epithelium of mice ventilated at a V_T _of 30 ml/kg for five hours. The increases of positive staining of phospho-ERK1/2 on airway epithelium were reduced via pharmacologic inhibition with PD98059. This confirmed that the PD98059 successfully inhibited ERK activation in high-V_T _ventilation both with and without hyperoxia.

**Figure 7:**
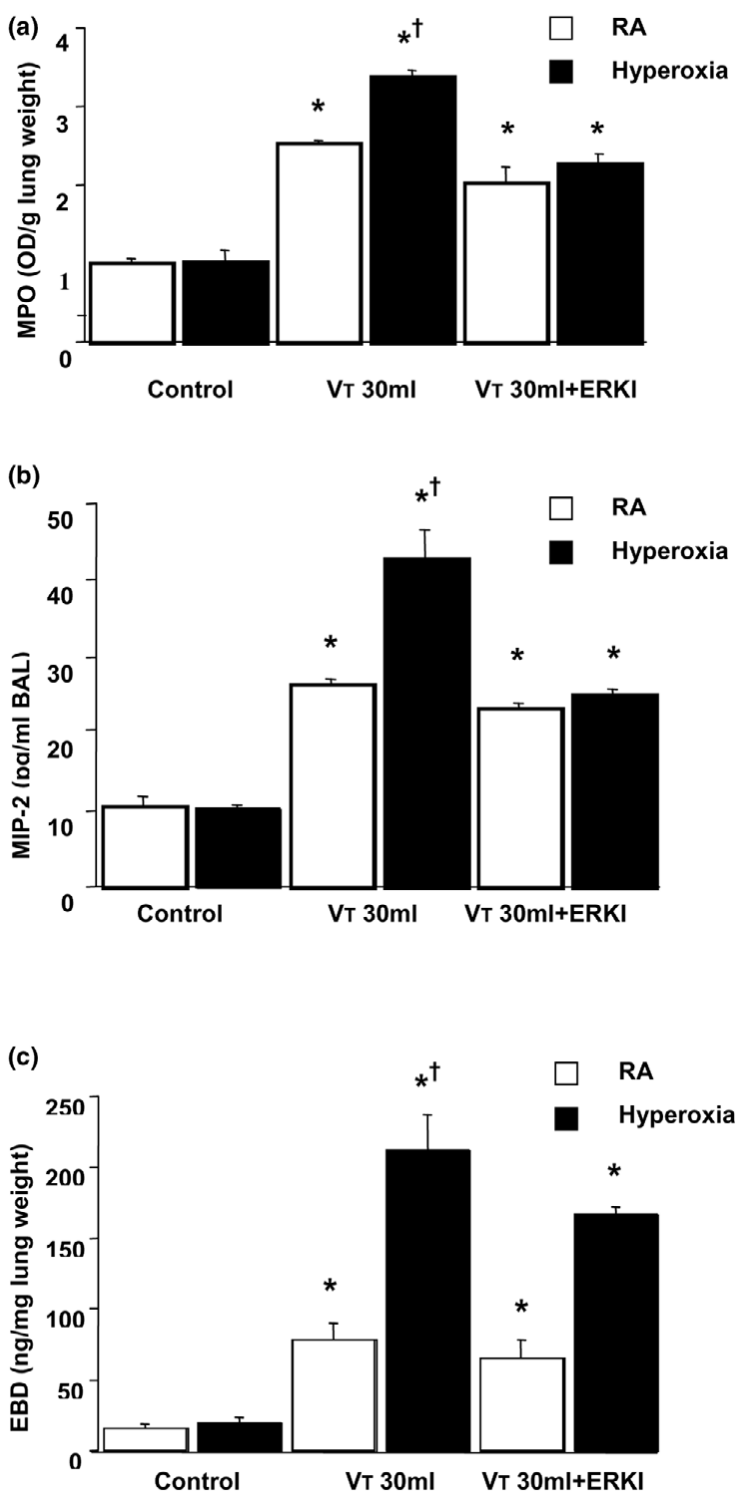
PD98059 reduced the effects of hyperoxia on stretch-induced neutrophil infiltration, macrophage inflammatory protein-2 (MIP-2) production, and microvascular leak. The mice ventilated at a tidal volume (V_T_) of 30 ml/kg (VT 30 ml) with and without hyperoxia were pretreated with 1 mg/kg PD98059 for 30 minutes. **(a) **Myeloperoxidase (MPO) assay of lung tissue (*n *= 5 per group). **(b) **MIP-2 production in bronchoalveolar lavage (BAL) fluid (*n *= 5 per group). **(c) **Evans blue dye (EBD) analysis of lung tissue (*n *= 5 per group). *p < 0.05 versus control, non-ventilated mice; †*p *< 0.05 versus all other groups. ERK, extracellular signal-regulated kinase; OD, optical density; RA, room air.

To determine the role of ERK1/2 activation in ventilator-induced neutrophil sequestration and upregulation of chemotactic factor with hyperoxia, we pretreated mice with specific ERK1/2 inhibitor (PD98059) for 30 minutes (Figure 7a,b). The increased MPO levels and the MIP-2 protein production were significantly reduced by using PD98059 in mice ventilated with hyperoxia at a V_T _of 30 ml/kg for five hours but not in mice ventilated with room air at a V_T _of 30 ml/kg. This suggested that the ERK1/2 pathway also contributed to neutrophil accumulation chemoattracted by MIP-2 in VILI during hyperoxia. Microvascular permeability, as measured by EBD, was significantly reduced by using PD98059 (Figure 7c).

### Hyperoxia increased lung stretch-induced apoptosis

**Figure 8:**
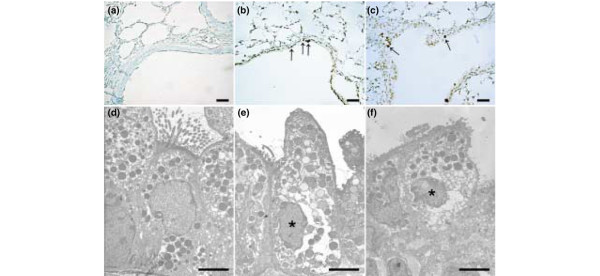
Apoptosis of airway epithelium during high-tidal volume (V_T_) ventilation with or without hyperoxia. Representative photomicrographs (×400) with TUNEL staining **(a-c) **(*n *= 5 per group) and electron micrographs **(d-f) **(*n *= 2 per group) of the lung sections after five hours of high-V_T _(30 ml/kg) mechanical ventilation without or with hyperoxia. **(a,d) **Control non-ventilated wild-type mice. **(b,e) **Wild-type mice ventilated at a V_T _of 30 ml/kg with room air. **(c,f) **Wild-type mice ventilated at a V_T _of 30 ml/kg with hyperoxia. Apoptotic cells are identified by asterisks or arrows. A dark brown DAB signal indicates positive staining of apoptotic cells, and shades of blue-green to greenish tan signify non-reactive cells. Highly condensed and fragmented heterochromatin of bronchial epithelial cells indicates apoptosis. Scale bars = 20 μm **(a-c)**, 4 μm **(d-f)**. DAB, diaminobenzidine; TUNEL, terminal deoxynucleotidyl transferase-mediated dUTP-biotin nick end-labeling.

We performed an apoptosis assay (TUNEL stain and electron microscopy) to determine the effects of hyperoxia on high-V_T _ventilation-induced apoptosis of airway epithelium. Apoptosis of alveolar epithelia was increased in mice with high-V_T _(30 ml/kg) mechanical ventilation for five hours while breathing room air (Figure 8b) or greater than 95% oxygen (hyperoxia) (Figure 8c) as compared with control, non-ventilated mice (Figure 8a). Apoptosis was further confirmed by electron micrographs that showed the characteristic nuclear condensation of bronchial epithelium in mice ventilated with high V_T _values while breathing room or greater than 95% oxygen (Figure 8e,f). This suggested that high-V_T _ventilation with room air or hyperoxia can induce lung injury by an apoptotic process.

### Inhibition of JNK or ERK reduced the effects of hyperoxia on high-V_T_-induced apoptosis

**Figure 9:**
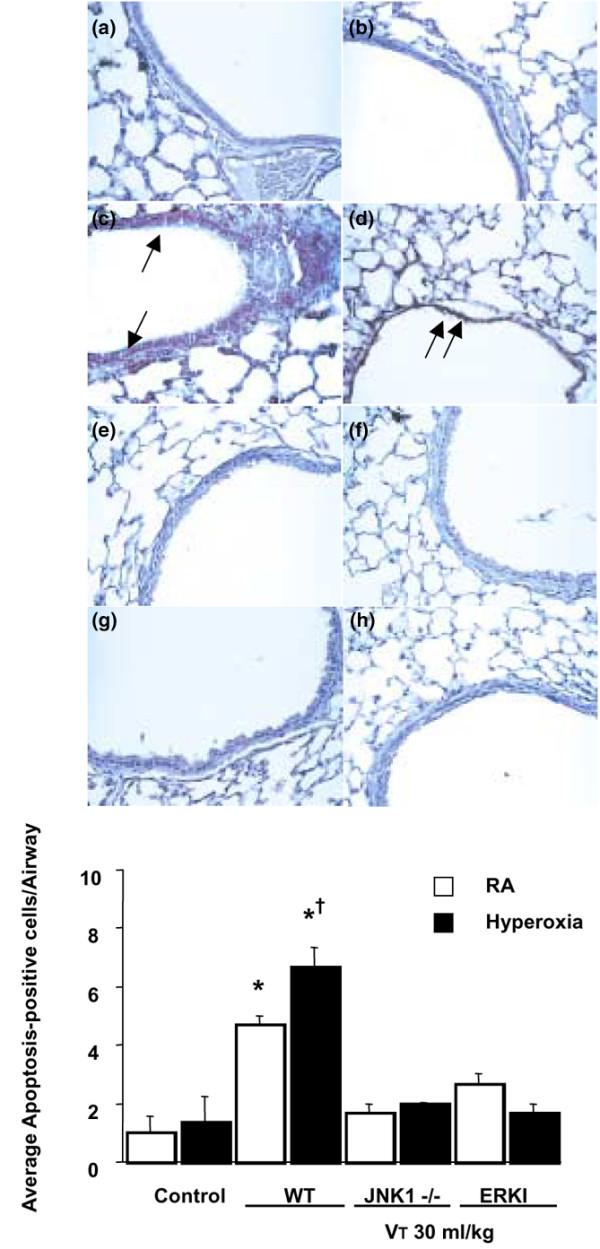
JNK knockout and PD98059-treated mice reduced hyperoxia-augmented, high-tidal volume (V_T_)-induced apoptosis of airway epithelium. The mice ventilated at a V_T _of 30 ml/kg with or without hyperoxia were pretreated with 1 mg/kg PD98059 for 30 minutes. Representative photomicrographs (×400) with cleaved PARP staining of the lung sections (*n *= 5 per group). **(a) **Control wild-type (WT) mice with room air (RA). **(b) **Control WT mice with hyperoxia. **(c) **WT mice ventilated at a V_T _of 30 ml/kg with RA. **(d) **WT mice ventilated at a V_T _of 30 ml/kg with hyperoxia. **(e) **JNK1^-/- ^mice ventilated at a V_T _of 30 ml/kg with RA. **(f) **JNK1^-/- ^mice ventilated at a V_T _of 30 ml/kg with hyperoxia. **(g) **WT mice pretreated with PD98059 and ventilated at a V_T _of 30 ml/kg with RA. **(h) **WT mice pretreated with PD98059 and ventilated at a V_T _of 30 ml/kg with hyperoxia. Positive staining of airway epithelia is identified by arrows. Cleaved PARP-positive cells were quantified as the average number of epithelial cells with dark brown DAB signals per bronchiole, which were counted from 10 randomly chosen bronchioles of each section (*n *= 5 per group). *p < 0.05 versus control, non-ventilated mice; †*p *< 0.05 versus ventilation in JNK1^-/- ^or with PD98059. DAB, diaminobenzidine; ERK, extracellular signal-regulated kinase; JNK, c-Jun NH_2_-terminal kinase; PARP, poly(ADP-ribose)polymerase.

To determine the roles of JNK and ERK1/2 activation in ventilator-induced epithelial apoptosis, we used homozygous JNK1 knockout mice and wild-type mice treated with the ERK1/2 inhibitor PD98059. To confirm programmed cell death by another method and to quantify the amount of apoptotic cells, we used the cleaved PARP antibody (89 kDa), a hallmark of apoptosis. The increased apoptosis, as measured by cleaved PARP activity, was reduced in JNK1 knockout mice and ERK1/2 activation with ERK1/2 inhibitor (PD98059) (Figure 9). This suggested that epithelial apoptosis induced by high-V_T _ventilation with room air or hyperoxia was dependent on the JNK and ERK pathways.

## Discussion

**Figure 10:**
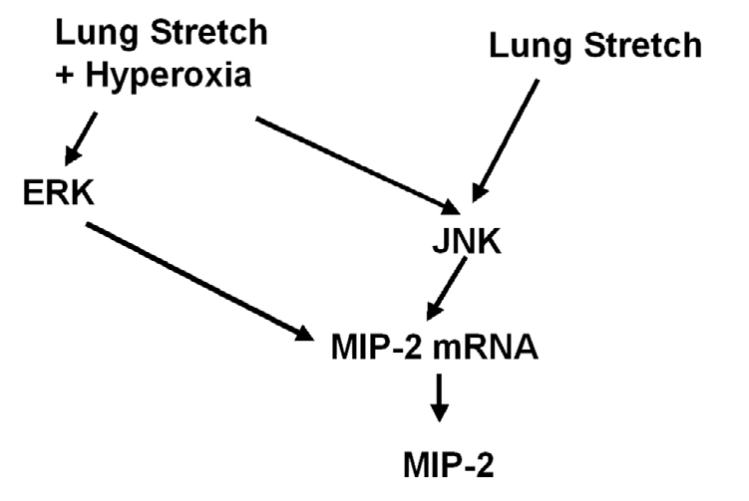
Differences in signaling pathway activation with lung stretch with mechanical ventilation with and without hyperoxia. Activation of the JNK pathway was involved in ventilator-induced neutrophil infiltration, cytokine production, and microvascular permeability with and without hyperoxia. Both ERK and JNK were involved in airway epithelial cell apoptosis with and without hyperoxia. The major difference in the signaling pathway activation was that ERK1/2 activation was involved in increased MIP-2 production and neutrophil infiltration only in mice exposed to high-tidal volume ventilation with hyperoxia, and not in mice exposed to high-tidal volume ventilation without hyperoxia. JNK activation was involved with and without hyperoxia. ERK, extracellular signal-regulated kinase; JNK, c-Jun NH_2_-terminal kinase; MIP-2, macrophage inflammatory protein-2.

We used hyperoxia as an oxidant stress model of VILI because it is commonly used in patients with ARDS. In this mouse model of VILI, we found that hyperoxia increased high-V_T_-induced neutrophil infiltration, MIP-2 protein production, microvascular permeability, and apoptosis in lung epithelial cells. Activation of the JNK pathway was involved in ventilator-induced neutrophil infiltration, cytokine production, and microvascular permeability with and without hyperoxia (Figures 1, 2, 3, 4, 5 and 9). Both ERK and JNK were involved in airway epithelial cell apoptosis with and without hyperoxia. However, ERK1/2 activation was involved in increased MIP-2 production and neutrophil infiltration only in mice exposed to high-V_T _ventilation with hyperoxia (Figures 3c, 6, 7, and 9). The differences in the activation of signaling pathways with lung stretch by mechanical ventilation with and without hyperoxia are depicted in Figure 10.

High-V_T _ventilation using hyperoxia has been shown in rat models to induce neutrophil migration into the alveoli and was dependent on the production of MIP-2, a chemoattractant belonging to the CXC family of cytokines [7,24]. Hyperoxia alone had minimal effects on both IL-8 and MIP-2 production [1,7]. In a previous study, we found that the addition of oxygen to rats ventilated at a V_T _of 20 ml/kg markedly increased the BAL content of neutrophils [7]. We also showed that hyperoxia increased high-V_T_-induced neutrophil sequestration and MIP-2 production (Figure 1) in the present study, but we went on to examine the pathways involved.

Hyperoxia may induce all three major MAPK (JNK, p38, and ERK1/2) pathways and transcription factors (AP-1 and NF-κB) in various *in vivo *(C57BL/6 mice) and *in vitro *(human A549 and mouse MLE-12 alveolar epithelial cells) experimental models [4,6,8,16,25-27]. Hyperoxia-induced lung injury has been found to be mediated by JNK, p38, and AP-1 pathways and may induce factors that inhibit downstream apoptotic events without significant phosphatidylserine translocation [6,8]. We found that hyperoxia increased high-V_T_-induced JNK activation, DNA-binding activity of AP-1, and MIP-2 mRNA expression after one hour of high-V_T _ventilation (Figures 2a, 2b, and 3a) and that these increases were even more elevated after five hours of ventilation (data not shown). We have previously shown a sustained increase in JNK activation over the span of five hours of ventilation in normoxia [2].

Others have shown that JNK1^-/- ^mice, after exposure to hyperoxia for 65 hours, have increased susceptibility to hyperoxia-induced epithelial cell apoptosis [16]. It is believed that transient activation of the JNK pathway by hyperoxia is protective, whereas prolonged exposure led to sustained JNK-mediated transcription of genes that are involved in apoptotic cell death [28,29]. For p38 phosphorylation, we found no significant difference between high-V_T_-ventilated mice with or without hyperoxia (Figure 3b). The p38 MAPK pathway may have contributed to post-transcriptional induction of IL-8 synthesis by stabilizing its mRNA via MAPK-activated protein kinase 2 and an AU-rich region [30], but this has not been explored in VILI.

Others have shown that ERK1/2, in addition to its involvement in the protective action of growth factors against cell death, played important roles as an upstream regulator of induced inflammatory mediators in response to stress and that inhibition of ERK1/2 reduced increases in lung injury parameters and neutrophil influx into the lungs [31]. Using rat and isolated mouse lung models, Uhlig and colleagues [10] found that the ERK pathway did not significantly contribute to the ventilator-induced releases of MIP-2 in spite of the increased expression of phopho-ERK1/2. In our study, inhibition of ERK1/2 with PD98059 (Figure 7) also did not affect high-V_T_-induced MIP-2 production or neutrophil sequestration while the mice were breathing room air. However, the increased neutrophil sequestration and MIP-2 production with VILI plus hyperoxia were reduced by PD98059. This suggested that JNK activation was necessary for lung stretch-induced cytokine production, but the augmentation of lung stretch-induced MIP-2 production by hyperoxia required the activation of both ERK1/2 and JNK pathways.

Previous studies have shown that hyperoxia may induce non-apoptotic or apoptotic epithelial cell death, depending on the species, cell-type specificities of hyperoxic insult, and culture condition [1-9]. Using human A549 alveolar epithelial cells and isolated mouse lung, others found that hyperoxia can directly cause lung injury by generation of reactive oxygen species and that mice exposed to hyperoxia will be more susceptible to the deleterious effects of high-V_T _ventilation [1,3]. Barazzone and colleagues [17] exposed mice to 100% oxygen for three or four days and found that both necrosis and apoptosis were involved in the cell death during hyperoxia. In our study, we found that high-V_T _ventilation with or without hyperoxia increased chromatin condensation of type II epithelia (characteristic of apoptosis) and damaged structures of the cytoplasm as compared with those of non-ventilated mice, JNK1^-/- ^mice, and wild-type mice pretreated with ERK inhibitor (Figure 8). Differentiating between apoptosis and necrosis can be difficult. We confirmed apoptosis using electron microscopy, TUNEL staining, and staining for cleaved PARP, suggesting that hyperoxia plus high-V_T _ventilation does induce cell death, at least in part, through an apoptotic pathway. Using JNK1^-/- ^mice and ERK inhibitor, we found that the apoptotic cell death decreased, suggesting the involvement of JNK and ERK in the apoptotic process of high-V_T _ventilation and hyperoxia (Figure 9).

ERK activation was involved in high-V_T _ventilator-induced apoptosis with and without hyperoxia but was involved in ventilator-induced lung inflammation only with hyperoxia (Figures 7 and 9). This suggests that the use of ERK inhibition as a therapeutic modality would be effective only in hyperoxia and not with the use of normoxia, whereas JNK inhibition would be effective no matter what the oxygen requirement were (Figure 10). We have previously shown that the pharmacological inhibition of JNK protected mice from VILI [20].

DNA-binding activity of NF-κB has been shown to increase after high-V_T _ventilation in *ex vivo *isolated mouse lung and *in vitro *human alveolar A549 epithelial cell studies [12,21]. Using normal human bronchial epithelial cells exposed to hyperoxia for five days, others found that NF-κB protected lung epithelial cells from hyperoxia-induced non-apoptotic cell death via NF-κB-mediated IκBα synthesis and by maintaining the levels of superoxide dismutase [4]. We found that DNA-binding activity of NF-κB was also increased after high-V_T _ventilation, but there were no significant effects of hyperoxia on its activation (Figure 2b). This suggested that NF-κB played a minor role in hyperoxia augmentation of VILI in comparison with AP-1.

The effects of high-V_T _ventilation and hyperoxia occur in the first few hours of VILI. In clinical practice after intubations, patients are often ventilated initially with higher V_T _values and 100% oxygen. Our data suggested that this practice may increase lung injury during the initial hours of ventilation.

## Conclusion

Using an *in vivo *mouse model, we found that hyperoxia increased high-V_T_-induced apoptotic and non-apoptotic epithelial cell injury and resulted in increased lung neutrophil sequestration and increased MIP-2 production, which were dependent, at least in part, on the JNK and ERK pathways. These data have added to the understanding of the effects of mechanical forces in the lung. In patients with severe ARDS, maintaining a low V_T _(6 ml/kg) and low levels of oxygen (FiO_2 _[fraction of inspired oxygen] of less than or equal to 0.5) is sometimes not possible. The inhibition of JNK and ERK1/2 may offer new treatment options for these patients.

## Key messages

• Hyperoxia augments VILI.

• Hyperoxia augmentation of VILI depends on JNK and ERK activation.

• Inhibition of JNK and ERK1/2 may offer new treatment options for patients with severe ARDS.

## Abbreviations

AP-1 = activator protein-1; ARDS = acute respiratory distress syndrome; BAL = bronchoalveolar lavage; DAB = diaminobenzidine; DIG = digoxigenin; EBD = Evans blue dye; EMSA = electrophoretic mobility shift assay; ERK = extracellular signal-regulated kinase; IL = interleukin; JNK = c-Jun NH_2_-terminal kinase; MAPK = mitogen-activated protein kinase; MIP-2 = macrophage inflammatory protein-2; MPO = myeloperoxidase; NF-κB = nuclear factor-kappa-B; NIH = National Institutes of Health; PARP = poly(ADP-ribose)polymerase; TMB = 3,3', 5,5'-tetramethylbenzidine; TUNEL = terminal deoxynucleotidyl transferase-mediated dUTP-biotin nick end-labeling; VILI = ventilator-induced lung injury; V_T _= tidal volume.

## Competing interests

The authors declare that they have no competing interests.

## Authors' contributions

L-FL and Y-SK collected and analyzed the data. DAQ, S-KL, and C-HL reviewed and coordinated the study. All authors read and approved the final manuscript.
